# Shelf Life Potential and the Fruit Cuticle: The Unexpected Player

**DOI:** 10.3389/fpls.2019.00770

**Published:** 2019-06-12

**Authors:** Isabel Lara, Antonio Heredia, Eva Domínguez

**Affiliations:** ^1^Unitat de Postcollita-XaRTA, AGROTÈCNIO, Departament de Química, Universitat de Lleida, Lleida, Spain; ^2^IHSM La Mayora, Departamento de Biología Molecular y Bioquímica, Universidad de Málaga, Málaga, Spain; ^3^IHSM La Mayora, Departamento de Mejora Genética y Biotecnología, Consejo Superior de Investigaciones Científicas, Málaga, Spain

**Keywords:** cuticle, fruit, mechanical properties, cell wall, post-harvest, shelf life, treatments, water loss

## Abstract

The plant cuticle is an extracellular barrier that protects the aerial, non-lignified parts of plants from the surrounding environment, and furthermore plays important functions in organ growth and development. The role of the cuticle in post-harvest quality of fruits is a topic currently driving a lot of interest since an increasing bulk of research data show its modulating influence on a number of important traits determining shelf life and storage potential, including water transpiration and fruit dehydration, susceptibility to rots, pests and disorders, and even firmness. Moreover, the properties of fruit cuticles keep evolving after harvest, and have also been shown to be highly responsive to the external conditions surrounding the fruit. Indeed, common post-harvest treatments will have an impact on cuticle integrity and performance that needs to be evaluated for a deeper understanding of changes in post-harvest quality. In this review, chemical and biophysical properties of fruit cuticles are summarized. An overview is also provided of post-harvest changes in cuticles and the effects thereupon of some post-harvest procedures, with the purpose of offering a comprehensive summary of currently available information. Identification of natural sources of variability in relevant quality traits would allow breeding for the improvement of post-harvest life of fruit commodities.

Give me juicy autumnal fruit, ripe and red from the orchard

                                                                                                                     -Walt Whitman

## Introduction

Being the interface between aerial plant organs and the surrounding biotic and abiotic conditions, the cuticle behaves as a barrier against drying, chemical attack, mechanical injuries, and microbial infection. Because the chemical nature of cuticle components is mainly lipophilic, this outer layer has been historically supposed to simply serve a protective role, particularly against dehydration. Yet growing experimental evidence accumulated along the last years indicate that fruit cuticles play additional major roles on the post-harvest storage potential of these commodities (reviewed in Lara et al., 2014; Martin and Rose, 2014).

In fruits, the cuticle is not only of importance during growth and development, but also during ripening and the post-harvest period. Ripening is a genetically programmed, finely-tuned phase of fruit development (Seymour et al., 2013). Ripening-related events include cell wall degradation and fruit softening, which renders fruits edible but also favors water loss and compromises storage potential and sensory quality of produce. The cuticle has been reported to exert a modulating role on several key quality traits of fruit commodities, which confers the study of cuticle a special interest in the field of post-harvest research. Cuticle properties and change dynamics after harvest are also economically relevant, since any deterioration during the harvesting period and subsequent storage either at the packing house, at the grocery store or at the consumer’s household translates to a significant amount of fruit waste.

Firmness is an important trait determining storage and shelf life potential of fruit produce. Beyond the pivotal influence of adequate firmness levels on fruit texture and hence on sensory quality, soft tissues are more prone to pests, rots and mechanical damage, which dramatically restricts commercial life of fruit and cause important economic losses. For roughly half a century of intensive research on ripening- and postharvest-related fruit softening, the main focus has been placed on cell wall metabolism (Goulao and Oliveira, 2008), generally ignoring other potentially involved factors. The unexpected and repeated observations that genetically suppressed expression of several ripening-related cell wall-modifying proteins often failed to diminish or to delay firmness loss significantly (Rose et al., 2003) led to the view of cell wall disassembly as the result of a close cooperation among many different proteins. In this scenario, some reports suggest that additional factors such as moisture loss may also play a role in firmness changes of fruit (Saladié et al., 2007; Paniagua et al., 2013), hence pointing to cuticle composition and architecture as promising research targets in post-harvest studies. Additionally, cuticle confers specific mechanical and physical properties to fruit surface, and its inner side interacts intimately with the underlying epidermal cell walls.

Some recent reviews have surveyed the literature on the specific characteristics of fruit cuticles (Lara et al., 2015) and on cuticle impact in fruit quality (Lara et al., 2014; Martin and Rose, 2014; Lara, 2018). To the best of our knowledge, though, no previous paper has amalgamated the chemical, biophysical and post-harvest-related aspects of fruit cuticles. This review summarizes and updates current information on the chemical and biophysical properties of fruit cuticles, and offers a survey of their reported impacts on relevant quality traits such as water loss, firmness, and susceptibility to infections. An overview of the so far few available studies on changes in fruit cuticles after harvest and in response to post-harvest procedures is also provided, with the aim of highlighting the high sensitivity of fruit cuticles to external conditions. Differences in chemical composition, physical properties, and post-harvest change patterns across fruit species, varieties and cultivars might underlie the large diversity in storage and shelf life potential detected. A better comprehension of these issues may help tailoring specific preservation and handling strategies, hence reducing food waste and improving economic returns.

## The Fruit Cuticle

**FIGURE 1:**
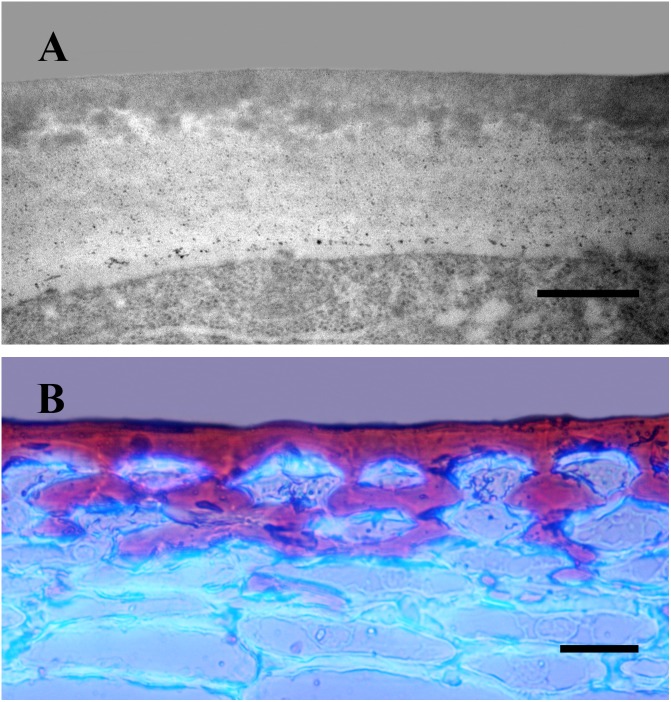
Cross-sections of tomato fruit epicarp. **(A)** Transmission electron micrograph of the outer epidermal cell wall of an immature fruit. Bar: 0.5 μm. **(B)** Mature tomato epicarp stained with Sudan IV and Calcofluor to visualize the cuticle and cell walls, respectively. Bar: 25 μm.

The cuticle can be regarded as an extreme modification of the outer epidermal cell wall. This is clearly observed as lipid material is deposited on the cell wall during cuticle development (Figure 1A). Thus, the non-cutinized region of the outer cell wall decreases during organ growth whereas the cuticle itself increases its thickness (Segado et al., 2016). Despite the progress in the last decades toward the understanding of the cuticle, it is still unknown how the cutin matrix is physically or chemically linked (or both) to the cell wall. Recently, it has been proposed that these two components could be interacting through specialized proteins (Jacq et al., 2017). The polysaccharide domain present within the cuticle is not clearly visible using conventional stains (Figure 1B) or transmission electron immunocytochemistry (Segado et al., 2016). Indeed, a partial degradation of the cutin matrix is needed to identify it Guzmán et al. (2014), an approach equivalent to those employed in cell wall analyses. This indicates that the lipid fraction of the cuticle is masking its polysaccharide domain. The use of confocal Raman microscopy, an infrared non-invasive technique, has allowed the *in vivo* identification of cell wall polysaccharides present in the *Arabidopsis thaliana* (L.) Heynh. cuticle (Prats Mateu et al., 2016). Therefore, the outer epidermal wall can be considered a highly asymmetric wall, from an inner purely cell wall region to an outer surface chiefly constituted of lipid material, with two opposite gradients, one of polysaccharides increasing toward the inside and another one of lipid material decreasing. Down-regulation of cell wall modifying enzymes in tomato (*Solanum lycopersicum* L.) fruit affected cuticle deposition (Vallarino et al., 2017; Jiang et al., 2019). Over the last years, an interesting interplay between the cuticle and the epidermal cell has been revealed. In this sense, cuticle disruption or impairment has been postulated to alter cell signaling thus affecting epidermal differentiation (Javelle et al., 2010). Several members of the Homeodomain leucine zipper IV (HD-ZIP IV) family of transcription factors are specifically expressed in the epidermal layer and have been shown to alter cuticle deposition as well as epidermal identity (Nadakuduti et al., 2012; Yan et al., 2018). However, the regulatory mechanism involved in cuticle deposition and epidermal identity still needs to be clarified.

Fruit cuticle analyses have been carried out in a significant number of crops. The cuticle has been shown to respond to environmental changes modifying its quantitative and qualitative composition together with its biophysical performance (Heredia-Guerrero et al., 2018). Nevertheless, exploration of different cultivars, varieties, naturally occurring mutants, related wild-species as well as segregant and mutagenized populations has uncovered a significant degree of variability in cuticle thickness, amount, chemical composition, biophysical properties, etc. (Domínguez et al., 2009a; Kosma et al., 2010; Yeats et al., 2012; Parsons et al., 2013; Verboven et al., 2013; Belge et al., 2014a,b; Petit et al., 2014; Fernández-Moreno et al., 2017; Leide et al., 2018). This variability clearly indicates that the cuticle can be subjected to plant breeding in order to improve some agronomically important traits (Petit et al., 2017). Over the last decades a number of genes have been identified to play a role in cuticle synthesis and deposition (Cohen et al., 2017). However, analyses of their expression profiles throughout development are limited and most of the work has been carried out in tomato fruit. From the post-harvest point of view, research efforts have been even more limited, and only a few studies have analyzed changes in the expression profile of cuticle-related genes during the post-harvest period (Wu et al., 2017; Carvajal et al., 2018; Belge et al., 2019; Li et al., 2019). This small handful of reports highlights substantial differences across species, cultivars and post-harvest procedures. Hence, much work is still needed to identify which of the cuticle-related genes play a significant role during post-harvest life, and how they are modulated by different storage conditions and treatments.

## Composition and Physiological Functions

**FIGURE 2:**
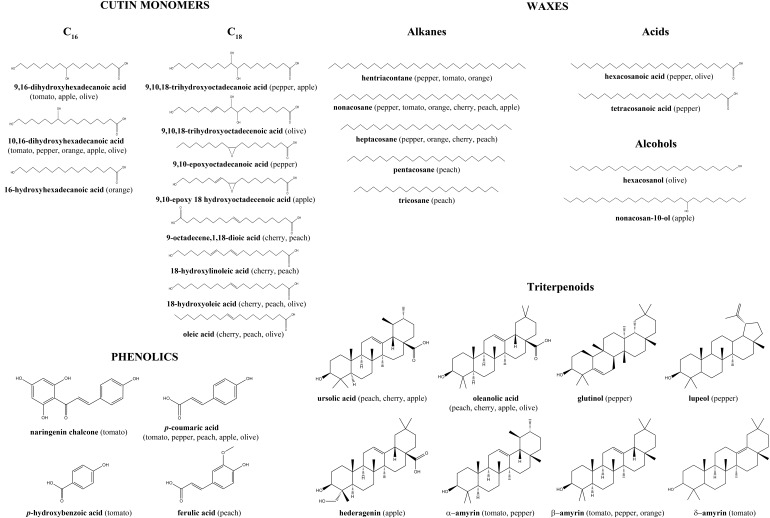
Main compounds present in the analyzed cuticles of fruit crops. Data taken from literature (Kosma et al., 2010; Fernández et al., 2011; Parsons et al., 2013; Belge et al., 2014a,b; España et al., 2014b; Wang et al., 2015; Huang et al., 2017; Leide et al., 2018).

The cuticle is mainly composed of a lipid matrix named cutin intertwined with a polysaccharide fraction derived from the epidermal cell wall. Cutin is usually more abundant than polysaccharides in the cuticle. In some fruit cuticles, an additional lipid matrix named cutan has been reported in combination with cutin (Domínguez et al., 2011b). Three classes of cutins have been identified depending on the chain length of the main fatty acids: C_16_, C_18_, or mixtures of both. Full chemical analyses of fruit cuticles have only been carried out in a limited number of species and the main identified components are shown in Figure 2 (for a detailed study see Lara et al., 2015). The 9(10),16-dihydroxyhexadecanoic acid is the main component of C_16_ cutin, while C_18_ cutins show more monomer variability including 18-hydroxy-9,10-epoxyoctadecanoic, 9-octadecene-1,18-dicarboxylic acid, 9,10,18-trihydroxyoctadecanoic acid and their derivatives. Chemical analysis of cutan composition has not yet been carried out in fruit cuticles and the main monomers remain to be identified. The polysaccharide domain has only been studied in tomato fruit cuticle rendering similar relative amounts of cellulose, hemicellulose, and pectin to those reported in tomato pericarp, thus reinforcing the idea that this domain is part of the primary cell wall (López-Casado et al., 2007).

Additional components of the cuticle are waxes and phenolics. Waxes are complex mixtures of very-long-chain (≥22 carbons) lipid compounds such as alkanes, alkenes, fatty alcohols, fatty acids and triterpenoids like ursolic acid, oleanolic acid, and amyrins (Figure 2). Wax abundance and composition can vary tremendously from one species to another. For example in fruits such as tomato, waxes are a minor component that constitutes around 5% of the cuticle whereas in grape (*Vitis vinifera* L.) or olive (*Olea europaea* L.) they can comprise 30–50% of the cuticle (Casado and Heredia, 1999; Domínguez et al., 2009a; Huang et al., 2017). Wax location within the cuticle contributes to its anisotropy since they can be present either deposited on the outer surface, epicuticular waxes, or embedded within the cutin matrix, intracuticular waxes. Cuticle phenolics have received in the past little attention although their presence has been reported in tomato, peach (*Prunus persica* (L.) Batsch), olive and apple (*Malus domestica* Borkh.) fruit cuticles (Fernández et al., 2011; España et al., 2014b; Huang et al., 2017; Leide et al., 2018). The main compounds identified are *p*-coumaric, *p*-hydroxybenzoic and ferulic acids and the flavonoid chalconaringenin (Figure 2), although a yet unidentified flavonoid fraction has been detected in peach cuticle (Fernández et al., 2011). This domain is responsible for the intrinsic fluorescence emission present in the cuticle (Fernández et al., 1999, 2011). Cinnamic acid derivatives have been found esterified to the cutin matrix, while flavonoids have been postulated to be distributed within the cutin matrix forming clusters (Domínguez et al., 2009b; Leide et al., 2018).

As the outermost plant barrier, the cuticle plays several protective functions (Domínguez et al., 2017) (Table 1). Its hydrophobic nature allows the cuticle to protect the plant against water loss and regulate the exchange of water, carbon dioxide and other solutes with the environment (Schreiber, 2010). It is also a thermo-regulator that is, acts as a buffer allowing the plant to maintain, within a range, its internal temperature despite changes in environmental conditions (Casado and Heredia, 2001). The cuticle protects plants from pathogen invasion not only acting as a barrier but also triggering several defense mechanisms (Aragón et al., 2017). Epicuticular waxes and phenolics have been shown to filter UV light while favoring light reflection, thus protecting cells from harmful UV light (Pfündel et al., 2006). In this sense, the flavonoid chalconaringenin contributes to the mature red color of tomato fruits (España et al., 2014a). Epicuticular waxes also participate in the physical appearance of fruits modifying its glossiness, although the cutin matrix has recently been shown to also contribute to fruit brightness (Ward and Nussinovitch, 1996; Petit et al., 2014). Moreover, the nanostructure and hydrophobic nature of epicuticular waxes contribute to the lotus effect, allowing the cuticle surface to remain clean and dry (Koch and Barthlott, 2009).

**Table 1:** Main cuticle properties identifying the main component responsible and their role.

Property	Component	Function
Permeability	Intra (epi) waxes	Reduce water loss from tissues
Hydration	Polysaccharides	Absorb water
	Waxes	Reduce water absorption from the environment
Light	Phenolics/flavonoids	Absorb UV-VIS light, contribute to fruit color
	Epicuticular waxes	Reflect light
Biomechanics: Fruit cracking Pest attack Herbivores	Polysaccharides Phenolics Waxes	Increase mechanical stiffness
Development	Cutin/waxes	Normal epidermal development Inhibits organ fusion
	Phenolics/cutin/waxes	Epidermal cell size
Thermal	Waxes/cutin	Act as a thermo-regulator
Gas exchange	Cuticle	Contribute to gas exchange
Self-cleaning	Epicuticular waxes	Surfaces remain clean and dry
Surface glossiness	Epicuticular waxes Cutin	Confer fruit brightness


The cuticle acts as a mechanically resistant barrier playing a very important structural role in organ integrity and protection against external or internal stresses either biotic or abiotic (Khanal and Knoche, 2017). Cell turgor pressure is the main internal mechanical stress, which is transmitted to the outer surface where the cuticle needs to withstand this pressure in order to maintain fruit integrity. This is crucial during ripening and post-harvest since cell wall degradation increases the amount of pressure directly exerted on the cuticle. The ability of the cuticle to yield under pressure explains its contribution to the control of organ growth. Over the last decade, the connection of the cuticle and the outer epidermal cell wall has led to a better understanding of its role during plant development. Its ability to act as a selective barrier not only prevents organ fusion and adhesion at early stages of development but also contributes to establish and maintain epidermal identity (Ingram and Nawrath, 2017). More recently, waxes have been postulated to act as signal molecules in several developmental processes related to cell growth and defense mechanisms (Nobusawa et al., 2013; Aragón et al., 2017).

Given the above-mentioned physical properties, it is clear that the cuticle has an impact on fruit external quality and can affect fruit market value (Lara et al., 2014). Changes in the cuticle can modify fruit glossiness or color hence affecting consumer’s preference, but they can also favor the appearance of cracking disorders, pest attacks or rapid water loss leading to fruit dehydration all of which will in turn affect the post-harvest shelf-life.

## Biophysical Properties

**FIGURE 3:**
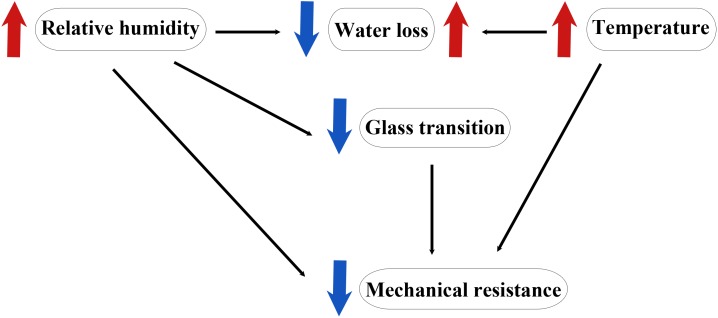
Simplified schematics of the interactions observed in the cuticle among temperature, relative humidity, and mechanical resistance.

Plants are surrounded by and need to cope with an ever-changing environment. In addition to variations in temperature, hydration, and light intensity plants also need to withstand mechanical stresses. The cuticle is thus a dynamic membrane that responds to environmental conditions. Water deficit, changes in relative humidity, temperature and light intensity and quality have been known to alter the amount, composition and/or thickness of the cuticle (Tattini et al., 2005; Léchaudel and Joas, 2007; Domínguez et al., 2012). In the case of fruits, it may be considered that this dependence on the environment ceases after harvesting, however this is not completely true. Currently, commodities go through storage and long distance travels therefore, they need to remain in their optimal conditions for longer periods. Understanding how the cuticle modulates its biophysical properties in response to changes in temperature, relative humidity and mechanical stress, either provided by the fruit’s own mass or by damage during transport and handling, is crucial to determine the best conditions to guarantee fruit quality. The three most studied biophysical properties of the cuticle have been thermal, mechanical, and hydric. These properties are strongly interconnected since changes in one affect the others (Figure 3). As it will be explained below, water modifies the thermal properties, temperature affects the water properties and both, water and temperature, modulate the biomechanical properties of the cuticle. It is therefore necessary to understand these interactions in order to identify the most appropriate storage conditions to enhance shelf-life.

### Hydric Properties

Water transport across the cuticle is of chief importance during the ripening and post-harvest periods. Two opposite directions of water movement can be considered, uptake from the surrounding environment and loss from internal tissues which causes fruit dehydration and desiccation. Given that the cuticle has much lower permeability, around 1000 times lower than cell walls, it is clear that the cuticle is the main barrier to water movement (Fernández et al., 2017). Water soluble stains, mainly Toluidine Blue O, have been employed as an indirect method to measure cuticle water permeability using intact fruits (Petit et al., 2014). Despite the useful information provided and the possibility to quantitate the amount of stained surface, a better approximation to fruit water permeability would be to measure the rate of fruit water loss under controlled conditions (Saladié et al., 2007; Petit et al., 2014). This could serve as an approximation to cuticle permeability in cases where the cuticle cannot be isolated and would also provide valuable information on the behavior of the whole fruit.

Water sorption studies have shown that isolated cuticles are able to incorporate up to 8% of its dry mass in water, depending on the composition and contribution of each cuticle fraction (Domínguez et al., 2011b). The polysaccharide domain is the main responsible for water uptake (Domínguez and Heredia, 1999). In mature peach, trichomes play a more important role in fruit water sorption than the cuticle itself, most probably due to the high proportion of polysaccharides present in peach trichomes compared to the cuticle (Fernández et al., 2011). At relative humidity above 50–60%, the water incorporated to the cuticle is no longer adsorbed to the surface of the cavities present inside the cuticle but forms clusters of free water that hinders transport across the cuticle (Luque et al., 1995; Reina et al., 2001). Accordingly, water loss kinetics has been shown to decrease with increasing relative humidity (Riederer et al., 2015). It is important to mention that cuticle water sorption shows hysteresis that is, once hydrated it is more difficult, more severe conditions are needed, to dehydrate the cuticle. This should be taken into consideration when fruit storage and packaging conditions are being contemplated. A thorough analysis of the litchi (*Litchi chinensis* Sonn.) and longan (*Dimocarpus longan* Lour.) fruit water properties has revealed some complex water barriers (Riederer et al., 2015). These fruits are characterized by a robust pericarp that acts as a protective structure limiting water loss from the fleshy aril. Post-harvest water loss uncovered the existence of two transpiration barriers represented by the cuticles layering the epicarp and endocarp cells, and two water reservoirs, the mesocarp and the aril. Thus, the kinetics of water loss showed a biphasic behavior with a high initial rate controlled by the outer cuticle and a lower equilibrium rate determined by both the outer and inner cuticles (Riederer et al., 2015).

Several attempts have been made to correlate cuticle thickness with water permeability with negative results (Riederer and Schreiber, 2001). Waxes, especially intracuticular ones, have long been known to be the main barrier to water movement and hence lower cuticle’s permeability whereas the cutin matrix does not seem to participate (Zeisler-Diehl et al., 2017). Indeed, wax removal causes a significant increase in cuticle permeability as well as in fruit water loss (Knoche et al., 2000; Kumar et al., 2018). Comparison of cuticle water permeability across different species has shown no correlation with the amount of waxes *per se* but with the amount of very-long-chain aliphatic compounds (Jetter and Riederer, 2016). A recent analysis of the barrier properties of waxes have demonstrated that, despite intracuticular waxes being commonly considered responsible for reducing water permeability, the picture is more complex. In fact, when intracuticular waxes are rich in alicyclic compounds (triterpenes, sterols, etc.), epicuticular waxes are important in determining water permeability (Jetter and Riederer, 2016). In accordance, analyses of fruit water loss during post-harvest in pepper (*Capsicum annuum* L.), orange (*Citrus sinensis* (L.) Osbeck) and cactus pear (*Opuntia ficus-indica* (L.) Mill.) showed no correlation with the total amount of epicuticular waxes present in the cuticle (Cajuste et al., 2010; López-Castaneda et al., 2010; Parsons et al., 2012). However, chemical analysis of waxes in pepper fruit found a strong positive correlation between water loss and the amount of alicyclic compounds and a negative one with the amount of alkanes and aliphatic compounds (Parsons et al., 2012). Moreover, in peach the highest rate of fruit water loss was obtained after wax but not trichome removal, indicating that, while trichomes were important for water sorption, water loss depended on waxes rather than on trichomes (Fernández et al., 2011).

Analysis of several tomato cuticle mutants showed no correlation between the amount of cutin and fruit water loss during post-harvest (Isaacson et al., 2009; Girard et al., 2012; Nadakuduti et al., 2012), in agreement with the fact that waxes are the ones playing a role in water permeability. Only in those instances where the reduction of cutin was accompanied with a decrease in wax coverage, a significant increase in water permeability was observed (Girard et al., 2012; Shi et al., 2013). In a similar fashion, down-regulation of a cell wall invertase in tomato affected cuticle deposition and increased the amount of waxes, thus reducing fruit water permeability (Vallarino et al., 2017). Purple tomatoes overexpressing two transcription factors involved in anthocyanin biosynthesis displayed reduced water permeability and extended shelf-life compared to the control red ones (Zhang et al., 2013). This reduction in permeability could be associated with the increased percentage of waxes detected in purple tomatoes (España et al., 2014a). The mutations *ripening inhibitor* (*rin*) and *non-ripening* (*nor*) are commercially employed to improve storage and shelf-life in tomato. They both encode transcription factors that have been shown to alter cuticle composition from early stages of development, despite these mutants were initially thought to only affect ripening (Kosma et al., 2010). They exhibited differences in the wax profile compared to the wild-type ‘Ailsa Craig’ and a significant increase in C_18_ cutin monomers. Similarly, higher amounts of some C_18_ cutin monomers and an increase in waxes have been detected in hanging tomatoes harboring different *nor* alleles (Saladié et al., 2007; Kumar et al., 2018). These hanging tomatoes are characterized by a reduced water loss rate during post-harvest.

### Thermal Properties

There is scarce information regarding the behavior of the cuticle under different temperatures and most of it comes from the study of the tomato fruit cuticle. The cuticle has high specific heat, which means that it is able to maintain temperature despite changes in the environment, and exhibits a glass transition within physiological temperature (Matas et al., 2004). This glass transition marks a shift between two physical stages, one rigid below the transition and a second one viscous and liquid-like above it. This transition has been attributed to the lipid fraction of the cuticle (Matas et al., 2004) and its physiological implications need to be considered during post-harvest. Thus, at low temperatures the cuticle is more rigid and restricts molecule movement across it whereas at higher temperatures it is more fluid, increases its volume and decreases its resistance to deformation favoring transport across the cuticle (Domínguez et al., 2011b). Hence, high temperatures increase water movement across the cuticle (Knoche et al., 2000). The hydric status of the cuticle also affects its thermal properties especially the glass transition. At high relative humidity the glass transition will appear at lower temperatures (Matas et al., 2004; Stark et al., 2008). Hence, structural changes of the cuticle at temperatures above the glass transition decrease its water barrier properties while at the same time water reduces the temperature at which this transition occurs, reinforcing the effect.

Unfortunately no studies of thermal properties during post-harvest of fruit commodities have been carried out. However, a few reports on changes in cuticle amounts or composition during cold storage exist for apple (Dong et al., 2012; Li et al., 2019), sweet cherry (*Prunus avium* (L.) L.) (Belge et al., 2014a), orange (Ding et al., 2018), and peach (Belge et al., 2019). Substantial differences in these modifications and their time-course, though, have been detected among species and cultivars. Heat treatments also reportedly induced alterations and rearrangements in fruit cuticle composition, structure or permeability (Jacobi and Gowanlock, 1995; Lurie et al., 1996; D’Hallewin et al., 1999; López-Castaneda et al., 2010; Belge et al., 2019). It would be worth examining how and to which level external temperature influences water movement across the cuticle.

### Mechanical Properties

The cuticle can be described as a composite biopolymer with a biphasic mechanical behavior. Thus, at low stresses the cuticle has an elastic performance with instant deformation and recovery of initial conditions once the stress has been removed. However, at higher stresses deformation is viscoelastic that is, time-dependent and mostly irreversible (Khanal and Knoche, 2017). The threshold between both behaviors is physiologically significant since it delimits the stress needed to permanently deform the cuticle. The lower this threshold the easier to deform the cuticle and the lower the force needed to break it. Hence, the biomechanical properties of the cuticle in ripe fruits and during post-harvest are relevant since cell walls are being degraded and much of the hydrostatic pressure is being transmitted directly to the cuticle. Analysis of the mechanical properties of the cuticle of developing fruits have shown that ripening is accompanied by an increase in stiffness and a reduction of its deformability (Knoche et al., 2004; España et al., 2014b). The contribution of each cuticle component to the overall mechanical behavior has only been studied in tomato fruit. In this sense, the cutin matrix gives the cuticle its ability to deform and therefore is responsible for the viscoelastic behavior (López-Casado et al., 2007). The other main component of the cuticle, the polysaccharide fraction, contributes to the elastic performance, reducing its deformation and increasing the breaking stress (López-Casado et al., 2007). Waxes, mainly intracuticular ones, stiffen the cuticle and, in some cases, they also increase the breaking stress (Khanal et al., 2013; Tsubaki et al., 2013). Phenolics, flavonoids in tomato fruit, play an important biomechanical role since they confer stiffness, increasing the breaking stress, reducing strain, and lengthening the elastic phase (España et al., 2014a). Thus, phenolics and waxes act as fillers restraining the ability of the cutin matrix to deform under low stresses. It should be mentioned that, in the tomato fruit cuticle, flavonoids are biomechanically more important than waxes or the more abundant polysaccharides.

Fruit cuticle biomechanics have been shown to vary widely even among cultivars of the same species (Tsubaki et al., 2012; Khanal and Knoche, 2017). Compilation of the available information on cuticle biomechanics for different species and organs allowed Khanal and Knoche (2017) to identify a relationship between cuticle thickness and some biomechanical properties. Hence, stiffness was positively and significantly related with thickness whereas deformation showed a negative and significant relation. Also, a positive relation between thickness and maximum force sustained was found, albeit it was not significant (Khanal and Knoche, 2017). These results substantiate a role of cuticle thickness as a modulator of the cuticular biomechanical properties at the fruit level. Together with the cuticle, epidermal cells have also been reported to play a role in fruit skin biomechanics (López-Casado et al., 2007). In several species, high epidermal cell density and cuticle thickness have been reported to reduce fruit cracking due to an increased mechanical resistance to deformation (Özgen et al., 2002; Ginzberg et al., 2014; Correia et al., 2018; Jiang et al., 2019). Fruit cracking, a disorder that affects several species of fleshy fruits, mostly occurs during ripening and/or post-harvest as consequence of the tissues being subjected to pressures higher than the mechanical resistance of their cell walls and cuticle. A relation between tomato fruit cracking, fruit internal pressure and the biomechanical properties of the cuticle has been established (Domínguez et al., 2012). In several *Ribes* L. species, plum (*Prunus domestica* L.), sweet cherry and grapes an increase in cuticle strain has been identified as one of the sources leading to microcracking (Knoche et al., 2004; Knoche and Peschel, 2007; Khanal et al., 2011; Becker and Knoche, 2012). Cuticle microcracking has been shown to increase water loss, hence having a detrimental effect on shelf-life.

Relative humidity and temperature are key modulators of the biomechanical properties of the cuticle (Domínguez et al., 2011a,b). An increase in temperature or relative humidity has a plasticizing effect on the cuticle biomechanics reducing the stiffness and breaking stress while increasing strain (Matas et al., 2005). In this sense, an increase in relative humidity from 40 to 80%, regardless temperature, caused a twofold decrease in cuticle stiffness and a 1.5-fold decrease in the stress needed to break the cuticle. On the other hand, a 1.5-fold decrease in these two parameters was observed when temperature was increased above 23–30°C, depending on the relative humidity (Matas et al., 2005). Combination of high relative humidity and increased temperature produces an even more detrimental effect on the biomechanical properties of the cuticle. Cuticle weakening in ripe fruits can cause severe economic losses since its ability to withstand fruit mass, pressure exerted by internal tissues or handling during harvesting and further post-harvest treatments will be compromised. This can lead to disorders such as cracking, russeting, skin discoloration, browning, or opportunistic fungal growth. Cherry tomato is a clear example of the relevance of environmental water activity on biomechanics during storage and transport. These fruits are marketed in plastic containers with pierces for ventilation thus avoiding water accumulation inside the container and reducing fruit cracking and fungal growth (Domínguez et al., 2011a). Despite the relevance of the mechanical properties of the cuticle for fruit quality, studies on their evolution during post-harvest are still missing.

## A Survey of Recent Findinds of Cuticle Impact on Fruit Quality

An increasingly large bulk of experimental evidence supports the physiological relevance of fruit cuticles and their impact on a range of economically important quality attributes. The optimization of post-harvest procedures will thus entail a better comprehension of cuticle functions and impact on shelf life and storage potential. A survey of published literature revealed a range of important attributes of fruit commodities likely impacted by cuticle properties, including transpirational water loss, proneness to infections and physiological disorders, firmness and pesticide retention (Lara et al., 2014). All these associations would hence require exhaustive examination.

Mass loss is a general physiological phenomenon after harvest. Transpirational water loss after fruit have been removed from the plant results in decreased turgor of the harvested commodity, with consequent detrimental effects on sensory quality (appearance, juiciness, texture) and on economic return of produce. Together with physiological water loss, tissue softening and rots are major issues limiting shelf-life and storage potential of fruit commodities. The role of fruit cuticle in the modulation of skin permeability to water has been discussed in the previous section. In this section, therefore, the focus will be placed preferentially on cuticle relationships with firmness, textural aspects, and susceptibility to rots.

### Relationship Between Cuticle Features and Fruit Texture

The cuticle has been generally viewed as a lipid wrap covering and water-proofing fruit surface. The loss of turgor has been hence regarded as a major mechanism through which the cuticle could be involved in ripening-related changes in fruit texture. Some reports have shown the relevance of this factor as a driver of the softening process. A study on the ‘*Delayed Fruit Deterioration*’ (*DFD*) tomato mutant, which undergoes minimal fruit softening and susceptibility to rots in spite of otherwise normal ripening, showed similar cell wall disassembly, loss of cell-to-cell adhesion and cell wall-related gene expression rates in comparison with the reference ‘Ailsa Craig’ cultivar (Saladié et al., 2007). Instead, *DFD* fruit displayed minimal transpiration water loss and comparatively high cell turgor indicating that, in addition to changes in cell wall metabolism, cuticle composition and architecture must also exert a key role on ripening-related fruit softening. Similar results were reported for blueberry (*Vaccinium corymbosum* L.), another climacteric fruit species. These fruit are picked after attaining full ripeness, when the main cell wall modifications are mostly completed (Vicente et al., 2007), but do however soften to a variable extent after harvest in spite of limited cell wall changes. Interestingly, non-destructive compression tests revealed that, within high ranges of mass loss, firmness loss of blueberry was directly related to this trait (Paniagua et al., 2013) and, in agreement, sealing the stem scar significantly reduced firmness loss (Moggia et al., 2017). Yet, softening and mass loss rates in blueberry fruit have been shown to be highly correlated to the ursolic acid content in the cuticle, indicating the relevance of the composition of cuticular waxes on this attribute (Moggia et al., 2016). Accordingly, after examining changes in the triterpenoid content of cuticular waxes in eight grape (*Vitis vinifera* L.) cultivars during fruit development, it was suggested that the arrest of oleanolic acid biosynthesis observed in ripe fruit may result in the modulation of the mechanical toughness of the cuticle and in decreased disease resistance (Pensec et al., 2014). Additional evidence has been reported as well on a role of cuticle composition on fruit softening in pepper (Maalekuu et al., 2005) and tomato (Bargel and Neinhuis, 2004; Kosma et al., 2010).

In addition to cuticle impacts on fruit turgor, its intimate interaction with epidermal cell walls should not be overlooked when considering a possible role on fruit texture. The cuticle should be rather understood as the lipidized, outermost region of epidermal cell walls, displaying differential chemical and structural features (Fernández et al., 2016; Guzmán-Delgado et al., 2016). The coordinated nature of the changes observed in the cuticle and the epidermal cell walls during the development of ‘Cascada’ tomato fruit indicates a close interaction between these two supramolecular structures, and therefore suggests that the cuticle should be interpreted within the context of the outer epidermal walls (Segado et al., 2016). Hence cuticle impact on fruit softening may be exerted not only through the regulation of water status but also through its role providing physical support. In this context, a recent study surveyed the contribution of apple peel to fruit firmness in a total of 65 apple cultivars (Costa, 2016). Mechanical profiles of fruit were generated by means of a texture analyser to characterize the mechanical performance of skin in each case, and suggested to be suitable tools for use in breeding programs for the selection of apple cultivars with enhanced post-harvest performance. No chemical or structural information was obtained in that study, and thus the question arises as to how the differences in mechanical profiles relate to cuticle and epidermis properties, which will require a close examination. Another recent work examined the incidence of peel splitting, a major physiological disorder affecting post-harvest quality of banana (*Musa acuminata* Colla) fruit after storage in saturating humidity conditions (Brat et al., 2016). Rheological determinations in fruit from ‘925’ and ‘Grande Naine,’ respectively a susceptible and a resistant cultivar, revealed that, even though peel resistance and elasticity were higher in ‘925’ than in ‘Grande Naine’ fruit, total epicuticular wax amount was lower in ‘925’ than in the resistant cultivar ‘Grande Naine’ (92 vs. 146 μg cm^-2^, respectively). Splitting incidence was thus hypothesized to be associated at least partially with osmotic peel-to-pulp water flux taking place at high relative humidity as a result of the higher sugar content in the pulp than in the peel. Biaxial tensile tests, reflecting skin strain during growth, showed that lower cracking susceptibility in ‘Regina’ as compared to ‘Burlat’ sweet cherries may arise from higher skin elasticity, which implies stiffer skin and higher resistance to extension. Skin stiffness and turgor decrease with ripening and increase with temperature, and the mechanical properties of skin cell walls are intensely affected by cell wall swelling. Further information can be obtained by comparative studies on melting- (MF) and nonmelting-flesh (NMF) peach genotypes, which differ in their patterns of firmness loss along ripening. The biomechanical properties of the exocarp and mesocarp of ‘Spring Crest’ and ‘Oro A’ peaches, respectively a MF and a NMF cultivar, were examined along with the amount and localization of flavonols and hydroxycinnamic acids (Onelli et al., 2015). Results suggested an association between the permeability and firmness of the epidermis and the presence of flavonoids and hydroxycinnamic acids. In samples of the MF cultivar ‘Spring Crest,’ the reaction to compression and probe penetration was almost exclusively ascribed to the epidermis, which served as a mechanical support to the pulp, concomitantly with lower loss of cell turgor and cell wall disassembly.

Furthermore, the close communication at the cuticle-epidermis interface might involve additional mechanisms for the link between cuticle properties and fruit texture. Published experimental results indicate that *endo*-polygalacturonase (*endo*-PG) is somehow involved in fruit softening. Interestingly, *endo*-PG activity was found to be required to achieve the melting flesh texture in peach fruit, characterized by wide apoplastic spaces and partially deflated mesocarp cells, but had no critical influence on firmness loss of fruit, changes of symplast/apoplast water status apparently being the main process through which fruit firmness is regulated in peach (Ghiani et al., 2011). Accordingly, suppression of *PG1*, the product of which is a homogalacturonan-cleaving *endo*-PG, had unexpected effects on water status and hypodermal anatomy of ‘Royal Gala’ apple fruit. Cells beneath the cuticle of PG1-suppressed fruit remained tightly packed, in parallel with less water loss and shriveling, suggesting an impact on fruit firmness and juiciness through the regulation of water status. Reduced transpirational water loss in the fruit appeared to be linked to changes in cell morphology, expansion, and packing density (Atkinson et al., 2012). The deposition of behenic acid in thinner surface of cell walls of the net tissue in three cultivars of netted melon (*Cucumis melo* L.) fruit has been suggested to aid the shaping of an effective barrier to moisture loss (Motomura et al., 2015).

### Relationship Between Cuticle Features and Susceptibility to Rots and Pests

Disease resistance of fruit will largely determine commercial shelf life of produce, especially in those species which are particularly prone to rots. In addition, due to ripening-associated firmness loss, fruit will become progressively susceptible to mechanical injury during storage, which will further facilitate infection development. Some opportunistic pathogens will take advantage of surface microcracks, wounds, or natural openings such as stomata or lenticels and, accordingly, the synthesis of new waxes induced by exogenous ethylene in ‘Navelate’ orange fruit reportedly conferred protection against infection by *Penicillium digitatum* (Pers.) Sacc. by providing a physical covering of vulnerable surface areas (Cajuste et al., 2010).

Other organisms will be able to directly cause a breach and penetrate the cuticle. Fruit-infecting structures can sometimes be highly species- and tissue-specific (Liao et al., 2012). Cuticular characteristics such as the amount, thickness, chemical composition and mechanical properties will determine to a significant extent the chance of such a breach. Cuticle thickness has been demonstrated a relevant factor determining resistance to fungal infections in apple (Konarska, 2012), cranberry (*Vaccinium oxycoccos* L.) (Özgen et al., 2002), stone fruit (Crisosto et al., 1997), and table grapes (Marois et al., 1986; Gabler et al., 2003), including those caused by *Monilia fructicola* (G. Winter) Honey and *Botrytis cinerea* Pers., albeit these and other published reports also support a role for particular cuticular constituents in modulating disease resistance. Interestingly, highly significant correlations have been reported between the resistance of grapevines to *B. cinerea* and relative impedance of cuticular waxes (Herzog et al., 2015), which suggests the suitability of this parameter as a phenotypic factor for the prediction of grapevine resistance to *B. cinerea*.

Contrasting observations have been published on the role of cuticular waxes in determining susceptibility to infections: whereas dewaxed grape berries showed increased proneness to *B. cinerea* infection (Marois et al., 1985), no relationship was shown between the major epicuticular wax components of apple fruit and the severity of the infection by *Peltaster fructicola* Johnson, Sutton et Hodges or *Leptodontidium elatius* (F. Mangenot) de Hoog (Belding et al., 2000). Alkane and triterpenoid constituents of the cuticular waxes of Asian pear (*Pyrus bretschneideri* Rehder) have inhibiting effects on spore germination and mycelial growth of *Alternaria alternata* (Fr.) Keissl. (Yin et al., 2011). More recently, cuticular wax and surface hydrophobicity have been suggested to be essential in facilitating *A. alternata* infection of ‘Zaosu’ Asian pear fruit through the regulation of growth and differentiation during the pre-penetration phase (Tang et al., 2017). A specific set of cuticular wax components, including fatty acids, alkanes, terpenes, indole derivatives, ketones, amides, phenols and steroids, were present in fractions obtained from cuticles of grapevine genotypes resistant to powdery mildew (*Erysiphe necator* Schwein) and showed high (over 75% germination inhibition) antifungal activity (Özer et al., 2017).

The waxy bloom layer has also been found to largely determine the oviposition preferences of *Eupoecilia ambiguella* and *Lobesia botrana*, two grapevine moths. Oleanolic acid underlined ovipositional preferences of *E. ambiguella* and *L. botrana*, with some contribution of minor components on the wax layer (Rid et al., 2018).

Other studies have pointed toward the relevance of cutin composition and phenolic compounds as factors determining susceptibility to infections (Comménil et al., 1997; Bostock et al., 1999; Gabler et al., 2005; Isaacson et al., 2009; Shi et al., 2013). The high resistance of ‘Bolinha’ peaches to infection by *M. fructicola* was related to high amounts of chlorogenic and caffeic acids on the fruit surface, which suppressed cutinase activity, required for cuticle penetration. Cinnamic and benzoic acid derivatives also suppressed the activity of cutinase in cultures of the fungus (Bostock et al., 1999). Tomato mutants displaying only 5–10% cutin as compared to the wild-type show increased pathogen susceptibility (Isaacson et al., 2009). High susceptibility of tomato fruit to infection by *Colletotrichum coccodes* (Wallr.) S. Hughes was associated to low contents of C_16_ cutin monomers and of benzoic and *trans*-coumaric acids (Shi et al., 2013).

## Changes in Fruit Cuticles After Harvest

Until very recently, post-harvest changes in fruit cuticles had received little interest. However, the bulk of published reports indicate that fruit cuticles keep evolving after harvest. However, though not abundant, these studies show that no common change patterns can be expected for different species or even cultivars (Table 2).

**Table 2:** A summary of reported changes in cuticle properties after harvest and in response to post-harvest procedures.

Botanical family	Fruit type	Ripening type	Cultivar	Treatment	Effects on cuticle	References
**Rosaceae**						
Apple (*Malus domestica* Borkh.)	Pome	Climacteric	‘Sturmer’	9 months at 3°C	No changes in wax coverage or composition	Morice and Shortland, 1973
			‘Granny Smith’ and ‘Dougherty’	9 months at 3°C	Increase in total waxes and fatty acids	
			‘Elstar’ and ‘Elshof’	2% O_2_ and <1% CO_2_ at 1°C for 8 months	Changes in wax properties and chemical composition, especially during shelf life. Increased wax ester hydrolysis with storage	Veraverbeke et al., 2001
			‘Jonagold’ and ‘Jonagored’	1% O_2_ and 2.5% CO_2_ at 1°C for 8 months	Hydrolysis of the ester fraction, with increased relative concentration of nonacosane and nonacosan-10-ol	
			‘Autumn Gold,’ ‘Royal Gala’	1 μL L^-1^ 1-MCP (18 h at -1°C) + 6 months at -1°C	Delayed development of some wax constituents in 1-MCP-treated fruit	Curry, 2008
			‘Red Fuji’	7 months at 0°C	Sharp decrease in *n*-alkane and total wax levels	Dong et al., 2012
				1 μL L^-1^ 1-MCP (24 h at 0°C) + 7 months at 0°C	Attenuated changes in particular wax compounds in treated fruits compared to controls	
			‘Starkrimson’	180 days at 0–1°C	Moderate, steady increase in surface wax density	Li et al., 2017, 2019
				500 mg L^-1^ ethephon + 180 days at 0–1°C	Increases in total waxes, alcohols, olefins, *n*-alkanes, fatty acids, and esters compared to controls. Increased wax density and accelerated wax crystal melting. Higher expression level of genes involved in VLCFA and alcohol synthesis.	
				1 μL L^-1^ 1-MCP + 180 days at 0–1°C	Inhibited increase in ester content compared to controls. Delayed wax density and wax crystal melting. Lower expression level of genes involved in VLCFA and alcohol synthesis in comparison to the controls.	
Sweet cherry (*Prunus avium* (L.) L.)	Drupe	Non-climacteric	‘Somerset’	3 days at 20°C	No significant differences in cuticle yields	Belge et al., 2014a
			‘Celeste’	3 days at 20°C	70% increase in cuticle loads. Increased triterpene and *n*-alkane amounts.	
			‘Somerset’ and ‘Celeste’	14 days at 0°C	Increases in total cuticle load and cutin content per surface unit. Cultivar-related differences in the evolution of compound types.	
			‘Hongdeng’	30 mM BABA (20°C, 10 min) + 5 days at 20°C	Smoother cuticle and more integrated structure of subepidermal cells in treated fruit	Wang et al., 2015
Peach (*Prunus persica* (L.) Batsch)	Drupe	Climacteric	‘October Sun’ (melting)	5 days at 20°C	No significant differences in cuticle yields	Belge et al., 2014b
				14 days at 0°C + 0/5 days at 20°C	Significant increase in total cuticle amount 0 and 5 days after cold storage. No difference in wax coverage upon removal from cold storage but noticeable increase after shelf life at 20°C. Significant augment in cutin loads after cold storage but no changes thereafter. Strong inhibition of *PpLipase, PpLACS1*, and *PpCER1* gene expression.	Belge et al., 2019
				30 kPa CO_2_ for 48 h + 14 days at 0°C	Similar total wax coverage, but lower acyclic to cyclic ratio in treated fruit due to differences in the relative contents of particular wax families. Higher total cutin amounts compared to controls.	
				Hot air (50°C, 45 min) + 14 days at 0°C	Higher cuticle amounts than the controls. Higher wax content but lower acyclic to cyclic ratios due to altered percentages of triterpenes, phytosterols, and fatty acids. Lower cutin amounts upon removal at day 0 after storage, but no differences with the controls after 5 days at 20°C.	
			‘Jesca’ (non-melting)	5 days at 20°C	25% increase in total cuticle, wax and cutin loads. Increased triterpene and *n*-alkane amounts.	Belge et al., 2014b
			‘October Sun’ and ‘Jesca’	1 mM MeJa (20°C, 3 min)	Substantial, cultivar-specific differences in total cuticle per surface area after cold storage.	Graell et al., 2018
Strawberry (*Fragaria × ananassa* (Duchesne ex Weston) Duchesne ex Rozier)	Etaerio	Non-climacteric	‘Camarosa’	PL pulses (2.4-47.8 J cm^-2^) + 8 days at 6°C	Well-defined layer of epicuticular waxes comparedto controls	Duarte-Molina et al., 2016
Asian pear (*Pyrus bretschneideri* Rehder; *Pyrus sinkiangensis* T.T.Yu.)	Pome	Climacteric	‘Kuerle,’ ‘Xuehua,’ ‘Yuluxiang’	7 months at 3°C	Decreased in total cuticular wax. Glossier wax crystal structures. Cultivar-related differences in change dynamics for different compound types. Concomitant modifications in the expression level of a range of cuticle-related genes.	Wu et al., 2017
**Solanaceae**						
Tomato (*Solanum lycopersicum* L.)	Berry	Climacteric	‘Ailsa Craig’	9 days at 20°C	Increased content of total waxes, *n*-alkanes, *n*-alkadienes, and amyrins Increased yield stress, decreased extensibility	Saladié et al., 2007
**Rutaceae**						
Orange (*Citrus sinensis* (L.) Osbeck)	Hesperidium	Non-climacteric	‘Navelate’	3 weeks at 22°C	No changes in soft epicuticular waxes	Cajuste et al., 2010
				2 μL L^-1^ ethylene	Higher wax contents, lower incidence of cracking, peel pitting, and fungal rots	
			‘Bingtang’	40 days at 4 or 25°C	Decreased total cutin content, but unchanged percentages of the different monomer types. Lower intracuticular wax content after storage at 4°C compared to 25°C.	Ding et al., 2018
**Ebenaceae**						
Persimmon (*Diospyros kaki* L.f.)	Berry	Climacteric	‘Fupingjianshi’ and ‘Ganmaokui’	12 days at 20°C	No differences in cuticle thickness, increased number or depth of microcracks	Meng et al., 2017
**Anacardiaceae**						
Mango (*Mangifera indica* L.)	Drupe	Climacteric	‘Keitt’	18 days at 20°C	Continuous, substantial cuticle deposition	Tafolla-Arellano et al., 2017
			‘Kent,’ ‘Tommy Atkins,’ ‘Manila,’ ‘Ataúlfo,’ ‘Criollo,’ ‘Manililla’	15 days at room temperature	Increased cuticle and wax deposition, with genotype-related differences well-correlated to storage potential	Camacho-Vázquez et al., 2019
**Cucurbitaceae**						
Zucchini (*Cucurbita pepo* L.)	Pepo	Non-climacteric	‘Sinatra’ and ‘Natura’	14 days at 4°C	Lowered expression level of genes of the fatty acid elongase complex	Carvajal et al., 2018
**Ericaceae**						
Blueberry (*Vaccinium corymbosum* L.)	Berry	Climacteric	‘Legacy’ and ‘Brighwell’	30 days at 4°C	Decrease in total wax content. Cultivar-specific variations in change dynamics	Chu et al., 2018


Total wax amount in fruit cuticles of ‘Ailsa Craig’ tomatoes increased significantly during 9 days of post-harvest ripening at 20°C, with strong increases in *n*-alkanes, *n*-alkadienes, and amyrins (Saladié et al., 2007). Although no significant changes were observed in total cutin amounts, significant quantitative modifications from the mature green (MG) to the red ripe (RR) stages were found for particular cutin monomers, as well as for total flavonoid levels. Concomitant ripening-associated changes in extensibility, yield stress and viscoelasticity of the isolated cuticles suggested a role of cuticular composition on the mechanical attributes of fruit. In contrast, when total and soft epicuticular waxes of ‘Navelate’ oranges were monitored over a 3-week period at 22°C after harvest, no significant differences were observed (Cajuste et al., 2010).

The dynamics of such modifications in cuticle composition have also been found to show significant cultivar-related variations. For instance, the evolution of fruit cuticle composition in ‘October Sun’ and ‘Jesca’ peaches, respectively a melting- and a non-melting cultivar, was compared after being kept at 20°C for 5 days subsequent to harvest (Belge et al., 2014b). Total cuticle yield per surface area remained at similar levels in ‘October Sun’ fruit, both at harvest and after 5 days at 20°C, while a 25% increase was observed for ‘Jesca’ fruit, which involved both cuticular waxes and cutin monomers. Similarly, whereas no significant differences were detected in fruit of ‘Somerset’ sweet cherry after being kept 3 days at 20°C following harvest, a strong (70%) increase in total cuticle loads was found for ‘Celeste’ cherries (Belge et al., 2014a). Both for ‘Jesca’ and ‘Celeste’ fruit, the most conspicuous increases observed after harvest were those in triterpenes and *n*-alkanes. Substantial differences in key fruit quality traits such as firmness and mass loss were observed in parallel, which may suggest a relationship between cuticular composition and the modulation of these attributes.

The changes in cell structure of fruit skin in two persimmon (*Diospyros kaki* L.f.) cultivars (‘Fupingjianshi’ and ‘Ganmaokui’) were studied at harvest and after 12 days of storage at ambient temperature (20°C) (Meng et al., 2017). Even though maturity stage at harvest was similar in both cases as shown by ethylene production and firmness levels, ripening patterns diverged after storage, ‘’Fupingjianshi’ exhibiting shorter storage potential than ‘Ganmaokui.’ Light microscopy observations showed no obvious differences in cuticle thickness, but the number and depth of microcracks increased noticeably after storage, particularly for ‘Fupingjianshi’ fruit, which also suffered more rapid firmness and mass loss rates. Compositional and structural differences possibly underlie these dissimilar change dynamics, but no compositional data were reported. In addition to cuticle appearance, both cultivars also showed differences in ethylene sensitivity, and significant cultivar-related differences were observed as well in the structural changes in the epidermis and hypodermis taking place during storage.

Transcriptomic analyses of ‘Keitt’ mango (*Mangifera indica* L.) fruit skin showed that the biosynthetic pathways leading to cutin production were constantly up-regulated during overripening at 20°C and 60–65% relative humidity for 18 days (Tafolla-Arellano et al., 2017). These analyses revealed continuous and substantial cuticle deposition throughout ripening and overripening, levels achieving as much as 2100 μg/cm^2^ by the end of the storage period. Accordingly, very recent ultrastructural and compositional studies of six mango cultivars (‘Kent,’ ‘Tommy Atkins,’ ‘Manila,’ ‘Ataúlfo,’ ‘Criollo,’ and ‘Manililla’) showed that total cuticle and wax deposition increased over 15 days of post-harvest shelf life (Camacho-Vázquez et al., 2019), although noticeable cultivar-to-cultivar differences were observed as to cuticle architecture and post-harvest change dynamics. The different mango cultivars assessed differed in key traits such as water transpiration rates and firmness. Although such differences may not be simply related to cuticle features, results indicated that those cultivars showing higher cuticle deposition generally exhibited lower percentage of transpiration mass loss, less visual deterioration and more resistance to fruit fly attack.

### A Summary of Reported Effects of Post-harvest Treatments on Fruit Cuticles

#### Cold Storage

The simplest post-harvest procedure is to store fruits under controlled temperature and humidity conditions. However, both factors are known to influence rheological and mechanical properties of fruit cuticles (Edelmann et al., 2005; Matas et al., 2005), and concomitant alterations in cuticle performance and functions are expected during and after post-harvest storage. For example, saturating humidity conditions (100% RH) substantially reduce peel resistance in banana, resulting in peel splitting (Brat et al., 2016). Moreover, significant changes in the amount of particular chemical compounds have been reported for fruit cuticle composition in a few species after cold storage.

Taken together, the existing reports illustrate the profound differences in the fate of fruit cuticle deposition and composition across different genotypes, and clearly indicate the inappropriateness of any generalization. Published investigations on apple fruit show substantial cultivar-to-cultivar variation in the modifications of cuticle composition after post-harvest cold exposure. Whereas no significant changes in wax coverage or composition were observed for ‘Sturmer’ apples after cold storage at 3°C for 9 months, considerable increases in total wax amount and abundance of fatty acids were found for ‘Granny Smith’ and ‘Dougherty’ fruit (Morice and Shortland, 1973). In contrast, long-term (7 months) storage at 2°C led to sharply decreased levels in *n*-alkanes and total waxes in ‘Red Fuji’ apples (Dong et al., 2012). More recently, wax density on the surface of ‘Starkrimson’ apple fruit has also been shown to increase, moderately but steadily, during storage at 0–1°C for up to 180 days (Li et al., 2019).

When sweet cherries of the cultivars ‘Celeste’ and ‘Somerset’ were kept at 0°C during 2 weeks, significant changes in cuticular wax composition were observed. Total cuticle load and cutin content per surface area increased significantly in fruit of both cultivars after cold storage. However, cultivar-related differences in the evolution of particular compound families were found (Belge et al., 2014a). ‘October Sun’ peach fruit, another species within the *Rosaceae* family, showed likewise an increase in total cuticle and cutin contents after storage at 0°C during 2 weeks (Belge et al., 2019). The expression of three genes involved in different steps of wax and cutin deposition, namely *PpLipase* (an acyl-CoA thioesterase related to cutin and wax production), *PpLACS1* and *PpCER1* (involved in the synthesis of waxes and cutin monomers from very long-chain fatty acids), was strongly inhibited during cold storage, and *PpCER1* expression levels did not recover even after fruit were transferred to 20°C. Although these modifications in gene expression levels were not in total agreement with the observed changes in the content of the corresponding cuticular components, data demonstrated that cuticle-related gene expression was intensely altered at the usual temperature commercially employed for the storage of peach fruit. Hence, a full comprehension of the molecular and biochemical mechanisms underlying cuticle biosynthesis will be required for the optimization of post-harvest quality of fruit.

Total cuticular wax in ‘Kuerle,’ ‘Xuehua,’ and ‘Yuluxiang’ Asian pear fruits decreased over storage at 3°C during 7 months compared to values at harvest, and wax crystal structures appeared glossier under scanning electron microscope observation (Wu et al., 2017). Yet, cultivar-related differences were observed in the change dynamics for particular wax compound types over cold storage. Transcriptomic analyses indicated that changes in the expression level of some genes potentially involved in wax biosynthesis were consistent with wax concentrations in each of the studied cultivars.

Expression levels of a range of genes belonging to the fatty acid elongase (FAE) complex, involved in the first steps of the biosynthesis of cuticular waxes, were also analyzed in fruit of ‘Sinatra’ and ‘Natura’ zucchini (*Cucurbita pepo* L.) stored at 4°C during 14 days (Carvajal et al., 2018). Similarly to the observations for ‘October Sun’ peaches, the expression of many of the genes analyzed decreased after cold storage, although no compositional analyses of cuticular waxes were undertaken.

Cuticular wax composition was also examined during post-harvest cold storage (30 days at 4°C) in ‘Legacy’ and ‘Brightwell,’ two blueberry cultivars. Although important quantitative and compositional differences were found between both cultivars and the dynamics of the compositional changes in wax was also cultivar-specific, total wax content decreased during cold storage in both cases (Chu et al., 2018).

Storage temperature has been also reported to affect the accumulation and crystal structure of cuticular waxes of ‘Bingtang’ oranges kept during 40 days at 4 or 25°C (Ding et al., 2018). Cutin content decreased during cold storage, although the relative amounts of the different monomer types did not change significantly at either temperature. After storage, the intracuticular wax content was lower in cold-stored fruit than in those samples kept at room temperature (6.74 vs. 10.06 μg cm^-2^, respectively). Time-course changes in each case were different too, and roughly paralleled those in the amount of terpenoids. Epicuticular waxes increased continuously along 40 days at 25°C, but decreased in cold-stored fruit in spite of a transient increase up to 30 days, concomitantly with the observed changes in the size of surface wax crystals.

#### Ethylene, Ethylene Releasers, and Ethylene Suppressors

Some reports suggest that ethylene may be required for the formation of at least some cuticular wax compounds. For example, the interruption of exposure of oranges to 2 μL L^-1^ ethylene led to the development of cracks in surface waxes in contrast to fruit submitted to a continuous treatment (Cajuste et al., 2010). Moreover, untreated controls displayed lower levels of total and soft waxes, in parallel to higher susceptibility to peel pitting and to infection by *Penicillium digitatum* (Pers.) Sacc., suggesting that ethylene may elicit or modulate protective mechanisms through changes in the fruit cuticle.

More information on the impact of ethylene on cuticular waxes is available for apple fruit. ‘Autumn Gold’ and ‘Royal Gala’ apples treated with 1-methylcyclopropene (1-MCP) and then submitted to cold storage for 6 months showed a delay in the development of some wax constituents (Curry, 2008). 1-MCP treatment attenuated the decrease in *n*-nonacosane and the increases in nonacosan-10-ol and nonacosan-10-one levels during long-term cold storage of ‘Red Fuji’ apples in comparison with untreated controls (Dong et al., 2012). Ethephon and 1-MCP treatments respectively increased and delayed the accumulation of cuticular waxes during cold storage of ‘Starkrimson’ apple fruit (Li et al., 2017). Ethephon positively regulated total wax coverage and the content of alcohols, olefins, *n*-alkanes, fatty acids and esters during cold storage at 0–1°C, while 1-MCP inhibited the increase in the amount of esters. Furthermore, the expression level of four key genes in apple wax biosynthesis were studied in response to post-harvest handling of samples, namely *MdCER4, MdWSD1* (both involved in the alcohol-forming pathways), *MdCER6* (a major gene for the synthesis of very long-chain fatty acids) and *MdMAH1* (a key gene in the final step of *n*-alkane biosynthesis) (Li et al., 2019). Neither ethephon nor 1-MCP induced detectable changes in *MdMAH1* expression levels. In contrast, ethephon increased the expression levels of *MdCER6, MdCER4* and *MdWSD1*, while on the contrary 1-MCP had an inhibitory effect thereupon. These recent studies thus provided experimental evidence that wax composition, at least in some apple cultivars, may be under ethylene control.

#### Modification of Storage Atmosphere

Controlled atmospheres allow extending commercial lifespan of fruit commodities. Yet such conditions are also known to induce remarkable changes in many complex metabolic pathways and to lead to profound changes in sensory and commercial attributes of fruit, some of them detrimental for consumer quality such as for instance those in aroma volatile emission (Lara, 2010). This is particularly important for commodities capable of enduring long storage periods such as apple or pear (*Pyrus communis* L.), which are commonly stored under very restrictive oxygen concentrations, and/or for long periods. It is therefore conceivable that, likewise temperature and relative humidity, storage atmosphere composition has an impact on fruit cuticle properties and therefore on cuticle-modulated fruit traits. Long-term controlled atmosphere under ultra-low oxygen affected indeed the structure and chemical composition of cuticular waxes of ‘Elstar,’ ‘Jonagold,’ and ‘Jonagored’ apple fruits during the subsequent shelf life period, the rates of wax ester hydrolysis increasing over prolonged storage (Veraverbeke et al., 2001).

A 30 kPa CO_2_ shock caused significant changes in cuticular wax and cutin composition in ‘October Sun’ peach fruit compared to those at harvest and in stored, untreated samples (Belge et al., 2019). Total wax coverage (g m^-2^) did not differ significantly in treated fruit in comparison with untreated samples or with the amounts at harvest upon removal from storage at 0°C for 2 weeks. However, profound differences were observed in the relative contents (% over total waxes) of particular wax families such as triterpenes, fatty acids and phytosterols, and the acyclic to cyclic ratio was significantly lower in CO_2_-treated than in untreated samples. In contrast to waxes, total cutin amounts per surface area increased significantly after cold storage in comparison with those at harvest, due to enhanced content of hydroxyacids. Additionally, CO_2_-treated samples displayed significantly higher amounts of α,ω-dicarboxylic acids than the controls and fruit at harvest. These observations indicate intense modifications in cuticle-related metabolism, deposition and structure, which are expected to have caused alterations in cuticular properties and hence on fruit properties. Indeed, mass and firmness loss were lower in treated fruit. These potential links would be worth a detailed examination in order to gain insight on the actual roles of individual cuticle components on fruit quality attributes.

#### Other Treatments

A few studies on the effects of other treatments on cuticle properties have also been reported. Peaches submitted to a hot air shock (50°C, 45 min) had significantly higher cuticle amounts (g m^-2^) than the controls after cold storage during 14 days, regardless of subsequent shelf life period (Belge et al., 2019), even though mass loss values were higher for treated fruit. Heat shock resulted in higher content of total cuticular waxes and in altered percentages of triterpene acids, phytosterols, and free fatty acids in comparison with the controls, which led to lower acyclic to cyclic compound ratios. Total cutin amounts were lower than those in the controls upon removal from cold storage, but no significant differences were observed after 5 days of shelf life at 20°C. Preliminary data on peach (‘October Sun’ and ‘Jesca’) fruit dipped in a 1 mM methyl jasmonate (MeJa) solution (20°C, 3 min) prior to storage at 0°C showed substantial differences in total cuticle yields (μg cm^-2^) between treated and untreated fruit after 2 weeks of cold storage (Graell et al., 2018). In spite of the observed differences in total cuticle loads, total wax percentage over total cuticle as well as the percentage of wax compound types over total waxes remained at similar levels irrespective of cultivar and treatment, suggesting modifications in the biosynthesis of cuticular waxes after harvest and storage. The consequences of these modifications on relevant fruit quality attributes will require a detailed consideration.

Pulsed light (PL) pulses in doses ranging 2.4–47.8 J/cm^2^ were applied to strawberries (*Fragaria* × *ananassa* (Duchesne ex Weston) Duchesne ex Rozier) prior to storage at 6°C during 8 days (Duarte-Molina et al., 2016). No compositional analyses were undertaken, but treated fruit showed a continuous and well-defined layer of epicuticular waxes as compared to controls, in parallel to 16–42% reduction in the incidence of post-harvest molds, significantly lowered fruit softening rates respecting day 0, better integrity of cell walls in the hypodermis, and increased deformability modulus, maximal rupture force and mechanical work values.

Post-harvest treatment of sweet cherries with the non-protein amino acid β-aminobutyric acid (BABA) delayed firmness loss and inhibited membrane leakage and malonylaldehyde accumulation (Wang et al., 2015). Treated fruit displayed higher content of cell wall polysaccharides and lower polygalacturonase and pectinmethylesterase activity levels. The chemical composition of fruit cuticles was not analyzed, but scanning electron microscopy observations showed that BABA-treated fruit had more smooth and well-defined cuticles and better integrated subepidermal cell structure than the controls.

## Conclusion

Over the last few years, the increasing interest in fruit cuticle properties, functions and roles on ripening- and postharvest-associated changes has translated into a growing number of published papers on these topics. Nevertheless, profound knowledge gaps still exist in the composition, architecture, physical properties and post-harvest changes of fruit cuticles. Current available information reveals noticeable differences across species and cultivars which need to be further explored in order to understand the relevance of these characteristics for post-harvest performance in each case. The understanding of the relationships between post-harvest traits and specific chemical components and/or structural features of fruit cuticles is still very preliminary. A meticulous investigation of the differences in fruit cuticular components at harvest and after storage among species and cultivars may lead to a better comprehension of the mechanisms underlying the wide variation in disease resistance and post-harvest potential, and thus to the possibility of tailoring post-harvest management for specific commodities. Additionally, biophysical analyses during post-harvest life are also needed in order to properly ascertain the role of the cuticle and cuticle components in shelf-life potential. Moreover, since information regarding the contribution of each cuticle component to water loss and to mechanical resistance of fruit is mainly limited to some model crops, further studies on non-model fruit species to support or delimit their role are much desirable.

## Author Contributions

ED and IL collected the literature mentioned in the manuscript. AH, ED, and IL conceptualized and wrote the manuscript.

## Conflict of Interest Statement

The authors declare that the research was conducted in the absence of any commercial or financial relationships that could be construed as a potential conflict of interest.
